# *snpGeneSets*: An *R* Package for Genome-Wide Study Annotation

**DOI:** 10.1534/g3.116.034694

**Published:** 2016-11-02

**Authors:** Hao Mei, Lianna Li, Fan Jiang, Jeannette Simino, Michael Griswold, Thomas Mosley, Shijian Liu

**Affiliations:** * Department of Data Science, University of Mississippi Medical Center, Jackson, Mississippi 39216; † Shanghai Children’s Medical Center, Shanghai Jiao Tong University School of Medicine, Shanghai, Shanghai Shi, China, 200023; ‡ Department of Biology, Tougaloo College, Jackson, Mississippi; § Department of Neurology, University of Mississippi Medical Center, Jackson, Mississippi 39174

**Keywords:** SNP, gene, gene set, genetic annotation

## Abstract

Genome-wide studies (GWS) of SNP associations and differential gene expressions have generated abundant results; next-generation sequencing technology has further boosted the number of variants and genes identified. Effective interpretation requires massive annotation and downstream analysis of these genome-wide results, a computationally challenging task. We developed the *snpGeneSets* package to simplify annotation and analysis of GWS results. Our package integrates local copies of knowledge bases for SNPs, genes, and gene sets, and implements wrapper functions in the *R* language to enable transparent access to low-level databases for efficient annotation of large genomic data. The package contains functions that execute three types of annotations: (1) genomic mapping annotation for SNPs and genes and functional annotation for gene sets; (2) bidirectional mapping between SNPs and genes, and genes and gene sets; and (3) calculation of gene effect measures from SNP associations and performance of gene set enrichment analyses to identify functional pathways. We applied *snpGeneSets* to type 2 diabetes (T2D) results from the NHGRI genome-wide association study (GWAS) catalog, a Finnish GWAS, and a genome-wide expression study (GWES). These studies demonstrate the usefulness of *snpGeneSets* for annotating and performing enrichment analysis of GWS results. The package is open-source, free, and can be downloaded at: https://www.umc.edu/biostats_software/.

GWS have generated abundant results in the past decade, with the advent of next-generation sequencing technologies boosting the number of findings. Two common GWS that contribute to these results are the GWAS, which tests phenotype-SNP associations, and the GWES, which examines differential gene expressions (Visscher *et al.* 2012; Rung and Brazma 2013). Before effectively interpreting GWS results, we must quickly and efficiently perform mass annotations. First, SNPs and genes must be mapped to their genetic positions. Then, all significant SNPs must be mapped to neighboring genes for functional attribution of individual GWAS findings. Lastly, we annotate the coordination of functions across multiple genes based on genetic knowledge bases and identification of functional pathways from the GWS results.

Functionally related genes, known as a gene set or pathway, typically act in concert to perform specific biological tasks influencing disease susceptibility and phenotype variation in the population (Hirschhorn 2009; Subramanian *et al.* 2005). After the primary analysis, researchers often investigate the genetic pathways enriched in the GWS results; the aim is to detect collective effects from groups of genes with shared biological function that may be obscured in individual tests of variants and genes during GWS (Hirschhorn 2009). These findings may shed light on the underlying biological processes. An integrated package, which allows rapid mapping between SNPs, genes, and gene sets, and implements functional pathway analysis, will play an important role in understanding GWS results and providing insight into the architecture of the disease.

SNP annotations can be extracted from the NCBI SNP database (dbSNP) (Sherry *et al.* 2001), a central public repository that describes all short genetic variants identified to date. Each SNP is assigned a reference “rs” ID, a widely accepted name that specifies a particular GWAS variant. This database uses the most recent genome build to assign map positions to all variants. However, published GWAS give variant and gene positions based on older genome builds, *e.g.*, NCBI36/hg18. It is challenging to quickly obtain updated annotation for all GWAS SNPs using online dbSNP searches. Efficient access to dbSNP, which enables fast remapping of a massive number of SNPs from the older genome build to the newest one (*e.g.*, GRCh37/hg19 or GRCh38/hg38), is therefore essential for accurate interpretation and downstream analysis of GWAS results.

Gene annotations can be sourced from Entrez Gene, a database that maintains records for genes found in organisms with completely sequenced genomes (Maglott *et al.* 2011). Every gene is assigned a unique identifier, map position, and official gene symbol approved by the HGNC (Eyre *et al.* 2006). The NCBI developed a data retrieval system, Entrez, that provides a user-friendly search of genetic annotations from dbSNP and gene databases (Geer and Sayers 2003). The UCSC Genome Browser is another interactive web-based resource that allows access to various types of genetic annotations (Fujita *et al.* 2011). Unfortunately, these web-based systems prevent the quick retrieval of a large number of annotations in a convenient data format for direct analysis.

Genes are the functional molecular units of genetics; their effects are measured to reveal genetic mechanisms underlying disease. GWES directly measures gene effects by testing differential gene expression associated with disease, whereas GWAS results must be further analyzed to indirectly measure gene effects through SNP associations. Each GWAS can generate over 1 million SNP associations, thus, it is a daunting task to quickly map the huge number of SNPs to genes and compute the gene effects (Peng *et al.* 2010). Before performing a pathway analysis to identify functionally related genes, a valid gene-based effect measure must be derived from the GWAS results. In addition, a pathway study relies on efficient access to, and analysis of, annotated gene sets from corresponding knowledge bases.

Various knowledge bases provide annotations for different types of gene sets: the Kyoto Encyclopedia of Genes and Genomes (KEGG) (Kanehisa *et al.* 2010) pathway database presents knowledge on the molecular interaction and reaction networks; the Gene Ontology (GO) (Harris *et al.* 2004) base uses controlled and structured vocabularies to define genes sets by their molecular functions, biological processes, and cellular components; and the Reactome pathway knowledge base provides molecular details of biological signals and cellular processes (Fabregat *et al.* 2016). In contrast to these specific knowledge bases, the MSigDB is regarded as a meta knowledge base; it not only generates annotation from publications but also collects gene set annotations from various knowledge bases, including KEGG, GO, and Reactome, through both manual curation and automatic computational means (Liberzon *et al.* 2011). Exploiting these knowledge bases can help identify functionally related genes for interpreting GWS results and performing downstream analysis (Subramanian *et al.* 2005; Mei *et al.* 2015; Hirschhorn 2009). These knowledge bases can be publicly accessed online or downloaded for local use. However, the annotation data are not well organized for direct analysis with GWS results. A software package that seamlessly integrates knowledge bases of gene set annotations for fast and convenient access is critically needed.

A common postanalysis of GWS results is the gene set enrichment study, which aims to identify whether genes from particular pathways are associated with a phenotype. Gene set enrichment analysis (Subramanian *et al.* 2005) was first developed to investigate pathway enrichment based on gene expression, but the statistical methods were extended to identify functional pathways based on SNP associations from GWAS (Wang *et al.* 2007). Since enrichment analyses use GWS results and do not require access to participant-level SNP and gene data, they can broaden the use of publicly available GWS results and enhance understanding of the GWS findings. However, the enrichment analysis requires comprehensive genome-wide annotations for SNPs, genes, and gene sets, which is time consuming and computationally burdensome, especially for GWAS results. An efficient software package with integrated flexible annotation has an important role in facilitating post-GWS enrichment analysis.

To facilitate the interpretation of GWS results and follow-up analyses, we developed a software package, *snpGeneSets*, which provides three main types of annotations: (1) genomic mapping annotation for SNPs and genes, along with functional annotation for gene sets; (2) bidirectional mapping between SNPs and genes, and between genes and gene sets; and (3) derivation of flexible gene effect measures from SNP associations and identification of pathways by enrichment analysis.

## Materials and Methods

**Figure 1:**
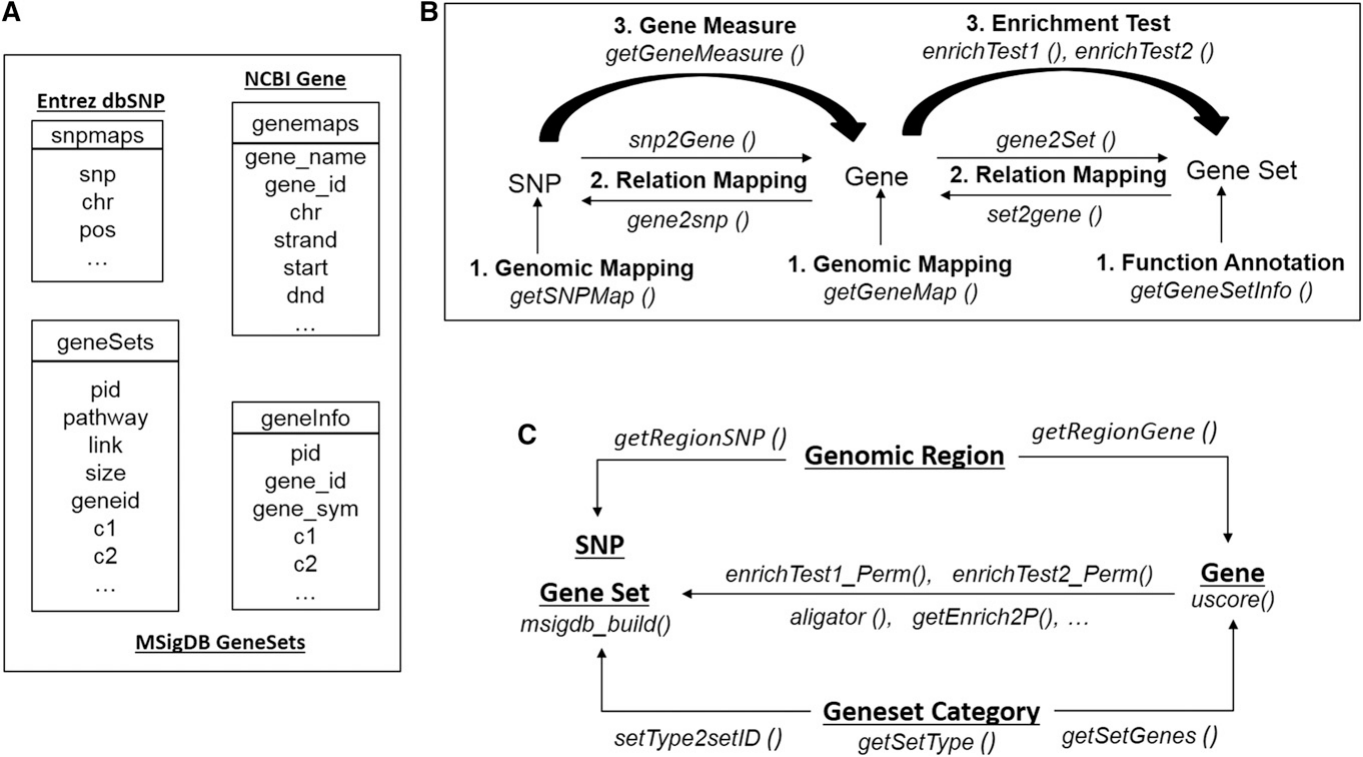
Systematic components of the *snpGeneSets* package. (A) Local genomic knowledge base: it parses the public NCBI dbSNP, Entrez Gene, and MSigDB databases to generate the SNP map (“snpmap”), gene map (“genemap”), gene sets (“geneSets”), and gene information (“geneInfo”) tables. (B) Three main annotations: (1) genomic mapping annotations for SNPs and genes, and functional annotation for gene sets; (2) relation mapping annotations between SNPs and genes, and between genes and gene sets; and (3) analysis-based annotation for measuring genes from SNP associations and testing gene set enrichment. (C) Auxiliary functions: they aim to support the first two major components (A and B), including identification of SNPs and genes from a defined genomic region, retrieval of genes and gene sets from a particular gene set category, permutation test and p-value calculations for gene set enrichment for genes, computation of the *U*-score for genes, and creation of a gene set database. MSigDB, Molecular Signatures Database; NCBI, National Center for Biotechnology Information; SNP, single nucleotide polymorphism.

The *snpGeneSets* package uses the *R* platform and the functions summarized in Figure 1 to support the interpretation and postanalysis of GWS results. The package invokes convenient wrapper functions to allow transparent access to the integrated genomic knowledge bases (Figure 1A), assign three main types of annotations (genomic mapping, relation mapping, and deriving gene measures and enrichment tests; Figure 1B), and implement auxiliary functions (Figure 1C) to support the annotations.

### Genomic knowledge bases

The knowledge bases are locally integrated into the *snpGeneSets* package and are built by parsing the online NCBI dbSNP (Sherry *et al.* 2001), Entrez Gene (Maglott *et al.* 2011), and MSigDB gene set (Liberzon *et al.* 2011) databases. The knowledge bases contain two tables titled “*snpmaps*” and “*genemaps*” for SNP and gene map annotations, respectively, based on the two most recent genome builds (GRCh37/hg19 and GRCh38/hg38). The SNP annotation includes all common variants and uncommon variants identified by the 1000 Genomes project. The gene annotation includes map positions of transcription start sites (TSS) and termination sites (TTS).

The genomic knowledge bases parse the MSigDB V4.0 gene sets to generate tables named “*geneSets*” and “*geneInfo*” (Figure 1A). The “*geneSets*” table provides annotation for every MSigDB gene set, including pathway name, link to the web site, functionally related component genes, and the size. Each gene set is assigned a unique *snpGeneSets* pathway ID (PID) to facilitate access. Gene sets are classified into different categories based on the methods or data sources that generate the annotation, *e.g.*, KEGG (Kanehisa *et al.* 2012), GO (Harris *et al.* 2004), or Reactome (Croft *et al.* 2014). In contrast to “*geneSets*,” the “*geneInfo*” table provides annotations for component genes instead of the gene set. The “*geneInfo*” table contains the Entrez gene ID, official gene symbol, the corresponding gene set, and the category.

The local genomic knowledge bases leverage the SQLite database management system. The *snpGeneSets* package integrates these knowledge bases and employs wrapper functions to permit transparent and seamless access to low-level databases and a large amount of genomic annotation using a simplified query. This package facilitates massive data integration while also being self-contained, serverless, maintenance-free, and platform-independent (with zero-configuration).

### Main annotations

Genomic mapping is the first type of annotation for SNPs, genes, and gene sets. The package includes the functions *getSNPMap()* and *getGeneMap()* to retrieve genomic mapping positions for a large amount of SNPs and genes, respectively (Figure 1B). The *getGeneSetInfo()* function returns the annotation for gene sets and component genes.

Relation mapping is the second type of annotation provided by *snpGeneSets*; its purpose is to identify bidirectional mapping relations between SNPs, genes, and gene sets (Figure 1B). Specifically, the function *snp2Gene()* simultaneously maps all genome-wide SNPs to genes based on flexibly defined gene boundaries, while the *gene2snp()* performs the reverse relation mapping and finds all annotated SNPs for one or more genes. The *gene2Set()* function identifies all gene sets that contain a particular gene and the *set2gene()* function does the reverse mapping to get all genes belonging to a particular gene set.

The third main component of *snpGeneSets* is the analysis-based annotation that reveals functional genetic pathways from GWS results (Figure 1B). This analysis is gene-centric, with the pathway identification dependent on the type of gene effect measure. The *getGeneMeasure()* function computes four gene effect measures from GWAS SNP associations (Table 1): (1) the *minP* method exploits the minimum p-value among SNPs in a gene as the measure of the gene effect; (2) the *2ndP* method selects the second strongest p-value as the gene measure (*2ndP*) [compared to the strongest SNP association in a gene, the second most strongly associated SNP is less likely to be influenced by outliers (Nam *et al.* 2010)]; (3) the *simP* method computes the Simes’ p-value to measure the gene effect using all SNP associations and an adjustment for the number of SNPs in the gene; and (4) the *fishP* method measures the gene effect by summarizing all SNP associations in a gene.

**Table 1:** Gene effect measures computed from the SNP association p-values

Method	Gene Measure	Description
minP	*P = p_(1)_*	The minimum p-value among SNPs in the gene
2ndP	*P = p_(2)_*	The second smallest p-value of SNPs in the gene
simP	*P = min_i_{K∙p_(i)_*/*i}*	Simes’ p-value adjusted for the number of SNPs
fishP	$P=pr\left(\chi_{df=2k}^{2}\geq -2\sum_{i=1}^{k}\log\left(p_{i}\right)\right)$	Fisher’s combined p-value

p_1_, p_2_, …, p_k_: the association p-values of *K* SNPs located in the same gene; *p*_(1)_ *≤ p*_(2)_ *≤ … ≤ p*_(_*_k_*_)_: the ordered association p-values of the K SNPs. $\chi_{df=2k}^{2}:$ a random variable that follows a chi-square distribution with 2 k degrees of freedom. SNP, single nucleotide polymorphism.

For GWES and other gene-based tests, *snpGeneSets* directly uses the gene expression p-value as the gene effect measure. To facilitate the interpretation and analysis, the *snpGeneSets* package converts GWS gene measures to uniform-scores (*U*-scores). Suppose *M_i_* is the gene measure for the *i*-th gene and *L* is the total number of genes. The *U*-score of the *i*-th gene is calculated as $U_{i}=\left(\sum_{j}I\left(M_{j}<M_{i}\right)+0.5\cdot \sum_{j}I\left(M_{j}=M_{i}\right)\right)/L.$ The *U*-score approximately follows a uniform distribution; the value estimates the percentage of genes with stronger effects than the examined gene.

In contrast to the mapping relations between genes and gene sets, the enrichment analysis identifies pathways enriched for genes with high ranked measures. The *snpGeneSets* package conducts two types of enrichment tests: (1) the candidate gene enrichment analysis (CGEA) compares candidate genes to all genes annotated in the knowledge base; (2) the uniform-score gene set analysis (USGSA) compares genes with *U*-scores meeting a criterion to all GWS genes. Both analyses apply a hypergeometric exact test to estimate the enrichment effects and obtain p-values directly from the distribution function. The enrichment test can be limited to a specific gene set category, *e.g.*, KEGG. The alternative (H_1_) hypotheses, parameter definitions, effect estimates, standard errors, and exact pathway p-values (*p_e_*) for the two tests are defined in Table 2. The *enrichTest1()* and *enrichTest2()* functions perform the CGEA (type 1) and USGSA (type 2) tests, respectively (Figure 1B).

**Table 2:** Two types of gene set enrichment tests

	Type 1 Test (CGEA)	Type 2 Test (USGSA)
H_1_ hypothesis	Candidate genes (Φ) are enriched in the tested gene set (Ω) of a particular category (*e.g.*, KEGG)	GWS genes with high ranked *U*-scores are enriched in the tested gene set (Ω) of a particular category (*e.g.*, KEGG)
Parameters	*L:* the number of genes (*G_i_: 1 ≤ i ≤ L*) in the gene set category (*e.g.*, KEGG).	*L:* the number of GWS genes (*G_i_: 1 ≤ i ≤ L*)
	$l=\sum_{i=1}^{L}I\left(G_{i}\in \Phi \right):$ the number of candidate genes	$l=\sum_{i=1}^{L}I\left(U_{i}\leq \alpha \right):$ the number of genes with *U*-scores in the top α (α = 5% in default)
	$S=\sum_{i=1}^{L}I\left(G_{i}\in \Omega \right):$ the gene set size	$S=\sum_{i=1}^{L}I\left(G_{i}\in \Omega \right):$ the gene set size
	$K=\sum_{i=1}^{L}I\left(G_{i}\in \text{Ω}\right)I\left(G_{i}\in \Phi \right):$ the number of genes in the gene set overlapping candidate genes	$K=\sum_{i=1}^{L}I\left(G_{i}\in \text{Ω}\right)I\left(U_{i}\leq \alpha \right):$ the number of genes in the gene set with *U*-scores in the top α (α = 5% in default)
Effect	*K/S-l/L*	*K/S-l/L*
SE	$\text{SE}=\left(l/L\right)\cdot \left(1-l/L\right)/\sqrt{S}$	$\text{SE}=\left(l/L\right)\cdot \left(1-l/L\right)/\sqrt{S}$
Exact p-value	$p_{e}=1-\sum_{i=0}^{K}\left(\begin{array}{c} S\\ i \end{array}\right)\left(\begin{array}{c} L-S\\ l-i \end{array}\right)/\left(\begin{array}{c} L\\ l \end{array}\right)$	$p_{e}=1-\sum_{i=0}^{K}\left(\begin{array}{c} S\\ i \end{array}\right)\left(\begin{array}{c} L-S\\ l-i \end{array}\right)/\left(\begin{array}{c} L\\ l \end{array}\right)$
*R* function	*enrichTest1()*	*enrichTest2()*

CGEA, candidate gene enrichment analysis; USGSA, uniform-score gene set analysis; KEGG, Kyoto Encyclopedia of Genes and Genomes; GWS, genome-wide study.

### Auxiliary functions

The *snpGeneSets* package also implements auxiliary functions to support the main annotations, local database creation, and enrichment analysis (Figure 1C). All the SNPs and genes located in a flexibly defined genomic region can be identified using the *getRegionSNP()* and *getRegionGene()* functions, respectively. Gene sets in the knowledge base are classified into different categories; the annotation for a particular category can be obtained using the *getSetType()* function. Mapping relations from a category to genes and gene sets can be characterized through the *getSetGenes()* and *setType2setID()* functions, respectively. These mappings, combined with the main annotations, can be applied to perform the enrichment test of a particular category, *e.g.*, KEGG.

Permutation tests, achieved through the functions *enrichTest1_Perm()* and *enrichTest2_Perm()*, obtain adjusted p-values for each pathway $p_{e}.$ To facilitate the fast calculation of adjusted p-values, a distribution table for $p_{e}$ was pregenerated based on 10,000 permutations for the USGSA (type 2) test. Thus, the *getEnrich2P()* function can generate permutation p-values instantly. Other auxiliary functions for annotation and postanalysis include *msigdb_build()* for building a local knowledge base of gene sets from MSigDB, *uscore()* for calculating genome-wide *U*-scores from gene effect measures, and *aligator()* for enrichment analysis of GWAS by the ALIGATOR method (Holmans *et al.* 2009).

### Data availability

The *snpGeneSets* package can be freely downloaded at https://www.umc.edu/biostats_software/. The GWAS catalog is available at https://www.ebi.ac.uk/gwas/. The T2D-GWAS result file supporting the annotation study of this article is available in the NCBI dbGaP under the accession number pha002839 (http://www.ncbi.nlm.nih.gov/projects/SNP/gViewer/gView.cgi?aid=2839). The T2D-GWES data can be identified at the NCBI GEO database with accession number of GDS3782 (http://www.ncbi.nlm.nih.gov/sites/GDSbrowser/?acc=GDS3782). The T2D-GWAS and T2D-GWES data can also be found at the *snpGeneSets* package.

## Results and Discussion

To demonstrate the usefulness of *snpGeneSets (version 1.12)*, we conducted annotation studies using GWS for T2D. We presented three applications using the NHGRI GWAS catalog, a Finnish GWAS, and a GEO GWES. These examples were performed on a Dell Latitude E6338 laptop equipped with a 2.90 GHz CPU (i7-3520M), 16.0 GB RAM memory, and a 64-bit Windows 7 Enterprise operating system.

### Genomic knowledge bases

The integrated local copies of annotation for SNPs, genes, and gene sets drive the expediency of the *snpGeneSets* package. The local knowledge bases contain mapping annotations for: 39,980,570 SNPs mapped to GRCh37 (both common variants with unique genomic positions from dbSNP and uncommon variants from the 1000 Genomes project); 14,270,004 common SNPs mapped to unique genomic positions in GRCh38; 39,814 and 41,409 gene transcripts mapped to GRCh37 and GRCh38, respectively; and 10,295 MSigDB gene sets composed of 32,364 genes classified into 20 categories.

### Annotation study of the NHGRI GWAS catalog for T2D

The NHGRI GWAS catalog provides a manually curated collection of published SNP-trait associations with p-values *<* 10^−5^ (Welter *et al.* 2014). We performed an annotation analysis of all T2D SNP associations included in the GWAS catalog (version updated 05/08/2016). We searched the trait “type 2 diabetes” and identified 349 SNP associations with p-values ranging from *[8E−75*, *9E−06]*; these associations represented 225 unique SNPs (Supplemental Material, Table S1). Using genome builds GRCh37 and GRCh38, snpGeneSets obtained positions for all SNPs in < 1 sec.

We mapped the T2D-associated SNPs to genes using build GRCh37 and boundaries 2 kb upstream of the TSS and 2 kb downstream of the TTE (Table S2); we identified 106 T2D genes from 134 unique SNPs. The *snpGeneSets* annotation (*e.g.*, SNP position, mapped gene official symbol, Entrez gene ID, and gene location) was compared to the gene information described in the original publication. For example, SNP *rs6712932* (PMID = 17668382) was reported as an intergenic variant but our annotation mapped it to *GPR45*; SNP *rs13424957* (PMID = 21347282) was reported as intergenic but mapped to a noncoding RNA gene (LOC101929633); *rs12304921* had no reported gene in published GWAS (PMID = 17554300) but mapped to *HIGD1C*; and *rs10190052* was located in *TMEM18* in the original publication (PMID = 24509480) but failed to map to any gene using our annotation (Table S1). In addition, *snpGeneSets* allows users to flexibly define gene boundaries for SNP-to-gene mapping.

We further performed CGEA (type 1) enrichment tests to identify pathways overrepresented by the T2D-mapped genes from the GWAS catalog. This analysis was limited to the KEGG category of gene sets and the 106 unique mapped genes which were inputted to the *enrichTest1()* function. The results are shown in Table S3. Of the 5267 genes included in the KEGG gene sets, 27 were T2D genes from the GWAS catalog. “*Maturity onset diabetes of the young*” with PID = 2866 (KEGG, http://www.genome.jp/dbget-bin/www_bget?hsa04950) was the only significant pathway with respective unadjusted and adjusted p-values of *6.44E−06* and 0 based on 1000 permutations (Table S3). The enrichment effect estimate suggested that T2D-mapped genes had a 15.49% higher probability of clustering in this pathway than random genes. This pathway is related to the monogenic form of T2D and contains 25 genes, including four T2D-mapped genes *(GCK*, *HNF4A*, *HNF1A*, and *HNF1B)*. Map annotations of the four genes using both genome builds (GRCh37 and GRCh38) are summarized in Table 3. *HNF4A*, *HNF1A*, and *HNF1B* encode transcription factors required for regulating the expression of several hepatic genes, while *GCK* encodes the enzyme glucokinase involved in the phosphorylation of glucose.

**Table 3:** Genomic mapping annotation for T2D genes from the GWAS catalog

Gene_ID	Gene_name	Full_name	Chr.	Start1 (bp)	End1 (bp)	Start2 (bp)	End2 (bp)	Strand
2645	GCK	Glucokinase	7	44,183,870	44,229,022	44,144,271	44,189,423	—
3172	HNF4A	Hepatocyte nuclear factor 4 α	20	42,984,441	43,061,485	44,355,801	44,432,845	+
6927	HNF1A	HNF1 homeobox A	12	121,415,861	121,440,315	120,978,058	121,002,512	+
6928	HNF1B	HNF1 homeobox B	17	36,046,434	36,105,096	37,686,431	37,745,105	—

“Start1” and “End1”, gene transcript start and end position based on GRCh37. “Start2” and “End2”, gene transcript start and end position based on GRCh38. Chr., chromosome.

### Annotation study of the T2D-GWAS

Results of the T2D-GWAS can be accessed from the NIH dbGaP database utilizing accession ID pha002839 (Scott *et al.* 2007; Tryka *et al.* 2014). This GWAS consisted of 1161 Finnish T2D cases and 1174 Finnish glucose-tolerant controls genotyped on the Illumina HumanHap300 BeadChip. SNP associations with T2D were tested by logistic regression under the additive genetic model. The GWAS results included 306,368 genotyped SNPs and a minimum p-value of 2.38E−06 at rs886374. There were 3 and 41 SNPs with p-values ≤ 1E−05 and 1E−04, respectively, and 0.1, 1.1, and 5.3% of SNPs with p-values ≤ 0.001, 0.01, and 0.05, respectively. We applied *snpGeneSets* to obtain SNP map annotation from the updated genome build. Annotation was provided for 306,252 SNPs from GRCh37/hg19 and 306,045 SNPs from GRCh38/hg38 in about 6 sec. The map annotation allowed quick access to accurate positions for all T2D-GWAS SNPs and identification of nearby genes for inferring SNP function.

Genomic mapping from SNPs to genes is the second type of annotation. We defined the gene boundaries as 2 kb upstream of the gene TSS and 2 kb downstream of the gene TTS. Using build GRCh37, the *snp2Gene()* function mapped 172,041 SNPs to 24,339 genes in about 10 min. 32% of mapped genes contained only one GWAS SNP. The number of genes and gene-mapped SNPs have a negative association; genes with more mapped SNPs tend to be bigger in size (Figure S1). The best SNP, *rs886374*, mapped to *SORCS2*.

**Figure 2:**
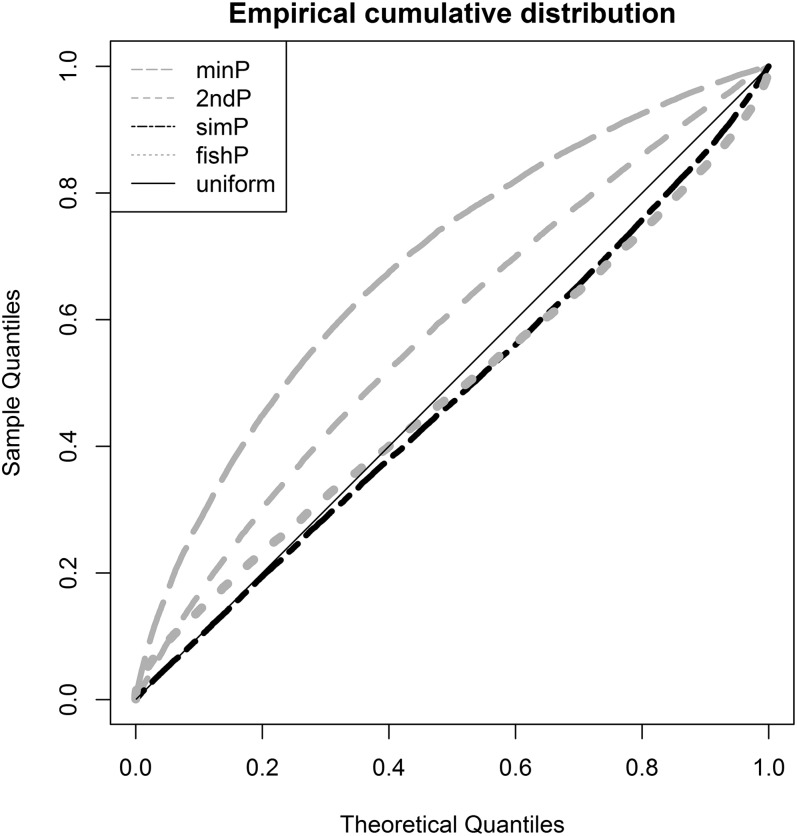
Q-Q plot of the gene effect measures. The observed sample quantiles of the *minP*, *2ndP*, *simP*, and *fishP* gene measures against the theoretical quantiles of the uniform distribution.

**Figure 3:**
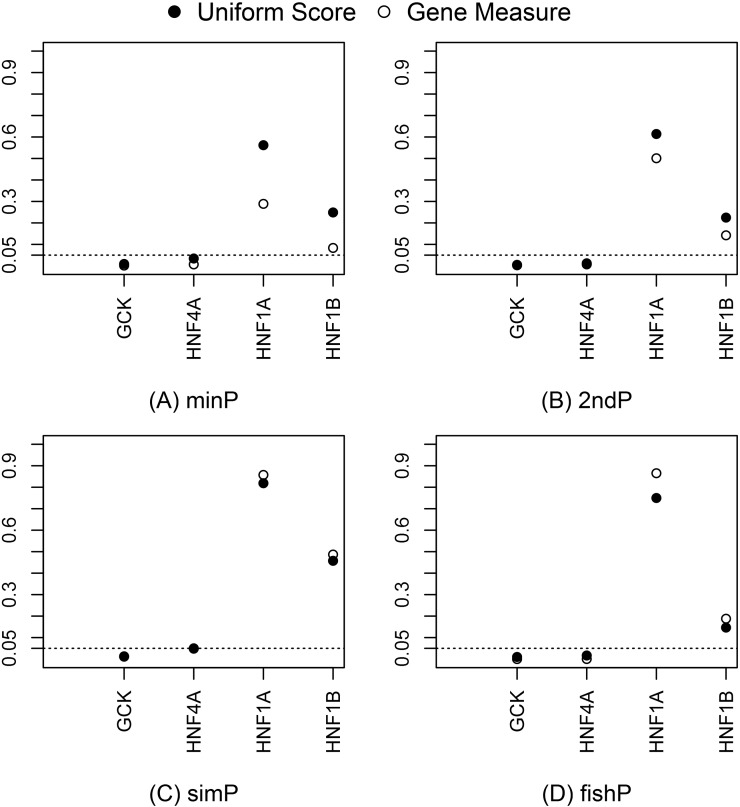
Gene measures and *U*-scores for *GCK*, *HNF4A*, *HNF1A*, and *HNF1B* using the type 2 diabetes genome-wide association study. The gene measure values and transformed *U*-scores for all four genes using (A) *minP*, (B) *2ndP*, (C) *simP*, and (D) *fishP*.

Measures and *U*-scores of gene effects were computed from SNP associations. The *minP*, *2ndP*, and *fishP* measures identified *SORCS2*, *TCF7L2*, and *LMO7* as the strongest genes, respectively; the *simP* measure identified microRNA genes, *MIR127*, *MIR136*, *MIR433*, and *MIR432*, as the strongest (Table 4). The distribution of the gene measure p was estimated by the percentage of genes with p ≤ α [*i.e.*, *Pr* (*P* ≤ α)]. The empirical cumulative distribution of different measures and the expected uniform distribution were plotted in Figure 2. The rank order for the best approximation of the uniform distribution is the *simP*, *fishP*, *2ndP*, and *minP* measure. The estimated *Pr* (*P* ≤ α) for α *=* 0.01/0.05/0.10 was 0.01/0.05/0.10, 0.04/0.09/0.14, 0.02/0.09/0.17, and 0.05/0.17/0.28 for the *simP*, *fishP*, *2ndP*, and *minP* measures, respectively. This demonstrated that gene identification depends on the gene effect measure used and different measures are not comparable due to heterogeneous distributions. In contrast, the *U*-score transforms all gene effect measures into uniform distributions and makes different measures comparable and interpretable on the same scale. We plotted the four measures and *U*-scores for the T2D-mapped genes (*GCK*, *HNF4A*, *HNF1A*, and *HNF1B*) from the GWAS catalog in Figure 3. *GCK* and *HNF4A* consistently ranked in the top 5% of all identified genes in the T2D-GWAS data. The respective *U*-score values corresponding to the *minP*, *2ndP*, *simP*, and *fishP* measures were 0.0089, 0.0048, 0.0127, and 0.0098 for *GCK* and 0.034, 0.012, 0.050, and 0.017 for *HNF4A*.

**Table 4:** The strongest gene effects identified in the Finnish T2D-GWAS

Method	Gene Measure	Gene_ID	Gene_name	Chr.	Strand	Start1 (bp)	End1 (bp)	Start2 (bp)	End2 (bp)
*minP*	2.38E−06	57537	SORCS2	4	+	71,94,374	7,744,564	7,192,647	7,742,837
*2ndP*	1.51E−05	6934	TCF7L2	10	+	114,709,978	114,927,437	112,950,219	113,167,678
*simP*	3.69E−05	406914	MIR127	14	+	101,349,316	101,349,412	100,882,979	100,883,075
		406927	MIR136	14	+	101,351,039	101,351,120	100,884,702	100,884,783
		574034	MIR433	14	+	101,348,223	101,348,315	100,881,886	100,881,978
		574451	MIR432	14	+	101,350,820	101,350,913	100,884,483	100,884,576
*fishP*	7.09E−17	4008	LMO7	13	+	76,194,570	76,434,006	75,620,434	75,859,870

“Start1” and “End1”, gene transcript start and end position based on GRCh37. “Start2” and “End2”, gene transcript start and end position based on GRCh38. Chr., chromosome.

We measured the effects of 102 T2D-mapped genes from the GWAS catalog in the T2D-GWAS data. *FishP*, *minP*, *2ndP*, and *simP*, respectively, identified 27.45, 19.61, 17.65, and 9.80% of genes with *U*-scores ≤ 0.05, which exceeded the expected percentage (5%) with p-values of 9.58E−07, 1.77E−04, 5.99E−04, and 0.054. Similar results were observed for *U*-scores ≤ 0.10, for which *fishP*, *minP*, *2ndP*, and *simP*, respectively identified 41.18, 32.35, 36.27, and 15.69% of the genes; the corresponding p-values for exceeding the expected percentage (10%) were 2.88E−09, 2.73E−06, 1.49E−07, and 0.060. The detailed results are shown in Table 5. Our analysis indicated that T2D-mapped genes from the GWAS catalog tended to have higher effect measures than random genes.

**Table 5:** Test of T2D-mapped genes from the GWAS catalog in the T2D-GWAS and T2D-GWES data

Data	*N*	Gene Measure	Mean1	Stat1	P1	Mean2	Stat2	P2
T2D-GWAS	102	*minP*	19.61%	3.70	1.77E−04	32.35%	4.80	2.73E−06
T2D-GWAS	102	*2ndP*	17.65%	3.33	5.99E−04	36.27%	5.49	1.49E−07
T2D-GWAS	102	*simP*	9.80%	1.62	0.054	15.69%	1.57	0.060
T2D-GWAS	102	*fishP*	27.45%	5.06	9.58E−07	41.18%	6.37	2.88E−09
T2D-GWES	86	p-value	12.79%	2.15	0.017	22.09%	2.69	0.004

*N*, the number of T2D-mapped genes from the GWAS catalog that were measured in the T2D-GWAS or T2D-GWES data; Mean1, percent of genes with *U*-scores ≤ 0.05; Stat1 and P1, the *t* statistic and p-value for testing Mean1 > 5%; Mean2, percent of genes with *U*-scores ≤ 0.10; Stat2 and P2, the *t* statistic and p-value for testing Mean2 > 10%. T2D, type 2 diabetes; GWAS, genome-wide association study; GWES, genome-wide expression study.

The four microRNA genes identified by the *simP* measure were absent from the KEGG category. However, the mapping relation annotation showed that *LMO7* and *TCF7L2* (identified by the *fishP* and *2ndP* gene measures, respectively) belong to the KEGG pathway “*cell-cell adherens junctions*” associated with diabetes (KEGG, http://www.genome.jp/dbget-bin/www_bget?hsa04520). In contrast to the mapping relation annotation, the enrichment analysis maps a set of genes to enriched pathways. The USGSA (type 2) test implemented by the *enrichTest2()* function examines enrichment of annotated pathways using genes with *U*-scores ≤ α. The T2D-GWAS enrichment analysis was performed on the KEGG category with α = 0.05, testing KEGG pathways enriched for genes with effect measures ranking in the top 5%. The negative logarithm of each pathway $p_{e}$ was shown in the Figure S2 and the enrichment results for the strongest pathway are summarized in Table 6. The *minP*, *2ndP*, and *fishP* measures consistently showed that “*Arrhythmogenic right ventricular cardiomyopathy*” with PID *=* 2901 (KEGG, http://www.genome.jp/dbget-bin/www_bget?hsa05412) was the strongest KEGG pathway with: enrichment effects of 17.8, 21.0, and 23.1%, respectively; unadjusted p-values ($p_{e}$) ≤ 4.63E−07; adjusted p-values (*p_table*) ≤ 0.0003 based on the pregenerated distribution table; and adjusted p-values (*p_perm*) *=* 0 based on 1000 permutation tests. The *simP* measure identified “*RIG-I-like receptor signaling pathway*” with PID *=* 2841 (KEGG, http://www.genome.jp/dbget-bin/www_bget?hsa04622) as the strongest pathway with an enrichment effect of 10.9% and *p_e_* of *7.65E−04*, but the adjusted p-value was > 0.05 (*p_table =* 0.26, *p_perm =* 0.31). Our results showed that the USGSA (type 2) test depends on the α value and the gene effect measure used to summarize SNP associations.

**Table 6:** The strongest KEGG pathway identified by the USGSA (type 2) enrichment analysis of the Finnish T2D-GWAS

Measure	Genes	PID	Size	SetGenes	Effect (%)	SE (%)	*p_e_*	*p_perm*	*p_table*
*minp*	289	2901	69	17	17.8	3.0	4.63E−07	0	0.0003
*2ndp*	274	2901	69	19	21.0	3.0	5.80E−09	0	0
*simp*	214	2841	50	8	10.9	3.1	7.65E−04	0.31	0.26
*fishp*	309	2901	69	21	23.1	3.1	1.28E−09	0	0

Genes: the number of GWAS genes with *U*-score ≤ 0.05 used for the enrichment analysis; PID, the pathway ID; “Size, the number of GWAS genes belonging to a Kyoto Encyclopedia of Genes and Genomes (KEGG) pathway; SetGenes, the number of GWAS genes belonging to a KEGG pathway with *U*-score ≤ 0.05; *p_e_*, unadjusted p-value; *p_perm*, the adjusted p-value based on 1000 permutations; *p_table*, the adjusted p-value based on the pregenerated distribution table. GWAS, genome-wide association study; GWES, genome-wide expression study.

### Annotation study of the T2D-GWES

The T2D-GWES data can be obtained from the GEO database (Accession ID: GDS3782) (Marselli *et al.* 2010; Barrett *et al.* 2011). This study assayed the abundance of gene transcripts in the pancreas using 10 control and 10 T2D human subjects (Marselli *et al.* 2010). In < 1 sec, the genomic mapping annotation identified map positions for 19,299 and 19,283 genes from GRCh37 and GRCh38, respectively. We tested the differential expression of each gene in T2D cases *vs.* controls through linear models and empirical Bayes methods implemented in the Limma package (Smyth 2004). The T2D-GWES results are integrated into the *snpGeneSets* package and can be accessed using the *load(T2DGWES)* function in *R*.

We directly measured the gene effects using the differential expression p-values and computed the *U*-scores by the auxiliary function *uscore()*. Of the T2D-mapped genes from the GWAS catalog, 86 genes had expression measured. Among these genes, 11 had *U*-scores ≤ 0.05, accounting for 12.79% of the T2D-mapped genes. The *t*-test showed that this significantly exceeded the expected percentage (5%) with p-value = 0.017. Similarly, 19 T2D-mapped genes, accounting for 22.09%, were found to have *U*-scores ≤ 0.10, which was significantly higher than the expected percentage (10%) with p-value *=* 0.004. These results are shown in Table 5. This analysis suggested that T2D-mapped genes from the GWAS catalog ranked significantly higher than random genes using differential expressions.

The USGSA (type 2) test was conducted on the KEGG category to identify pathways enriched for differentially expressed genes. After adjusting for multiple testing by permutation, no KEGG pathway attained significance using α = 0.05 (results not shown). If we expanded the analysis to all categories, the strongest gene set was “*Neighborhood of SPINK1*” with PID = 2700 (MSigDB, http://software.broadinstitute.org/gsea/msigdb/cards/GNF2_SPINK1), which had *U*-scores ≤ 0.05 for 70% of the component gene*s*, an enrichment effect = 0.65, an unadjusted p-value = 2.40E−16, and a permutation adjusted p-value < 1E−5. The SPINK1 gene set originated from the mining of coexpressed genes (Liberzon *et al.* 2011) and belongs to the computational category. This gene set contains 27 component genes, most of which encode proteins secreted by the pancreas; the gene mapping annotations based on GRCh37 are summarized in Table S4. However, the pathway function is not certain because the gene set was computationally generated. Therefore, we performed the CGEA (type 1) test on the component genes and attempted to map the SPINK1 gene set to KEGG pathways. Our analysis showed that the KEGG “*Glycerolipid metabolism*” pathway with PID = 2756 (KEGG, http://www.genome.jp/dbget-bin/www_bget?hsa00561) was significant with unadjusted and adjusted p-values of 8.05E−07 and < 1E−3, respectively. Glycerolipid metabolism is involved in the pathogenesis of T2D (Prentki and Madiraju 2008). Thus, annotation studies of genome-wide expression data using *snpGeneSets* can enhance the understanding of an established pathway (*e.g.*, KEGG “*Glycerolipid metabolism*”) and help to identify pathway functions of annotated gene sets (*e.g.*, *SPINK1* and its neighborhood genes in the T2D pathogenesis).

### Features and comparison between other tools and packages

Various tools and packages are currently available for genetic annotation and postanalysis of GWS data. The NCBI dbSNP (Sherry *et al.* 2001), Entrez Gene, and MSigDB (Liberzon *et al.* 2011) databases provide comprehensive and specialized annotation for SNPs, genes, and biological pathways, respectively. The UCSC Genome Browser (Fujita *et al.* 2011) and Ensembl BioMart (Kinsella *et al.* 2011) facilitate the access to these annotations by a user-friendly interactive web search. Particular packages, *e.g.*, GSEA (Subramanian *et al.* 2005) and MAGENTA (Segre *et al.* 2010), analyze gene set enrichment for differential gene expressions and SNP associations. Unlike these tools and packages, *snpGeneSets* integrates heterogeneous annotation data from different sources, supports fast and efficient annotation-based studies on large scale genomic data, and simplifies post-GWS annotation and analysis in a unified platform. This is much more efficient than using multiple tools to access genetic annotations of SNPs, genes, and pathways, perform relation mapping among them, and conduct enrichment analysis.

The *snpGeneSets* package is focused on SNPs, genes, and pathways. Many GWAS-identified SNPs lie within noncoding genomic regions; annotations for noncoding variants and epigenetic factors, including transcription factor binding sites, DNA methylation sites, and histone modification regions, will further enhance post-GWS annotation and analysis. Cscan (Zambelli *et al.* 2012), HaploReg (Ward and Kellis 2012), and RegulomeDB (Boyle *et al.* 2012) identify epigenetic factors related to a list of SNPs or genes. The ENCODE ChIP-Seq Significance Tool identifies the transcription factors enriched in genes based on public ENCODE data (Auerbach *et al.* 2013). *snpGeneSets* can be used with these packages and tools to provide a comprehensive understanding of genome-wide SNP association and expression findings and perform effective post-GWS analysis.

### Conclusions

We developed an open-source *R* package named *snpGeneSets* that annotates and conducts enrichment analysis on GWS results. The package integrates local copies of the annotation knowledge bases for SNPs, genes, and gene sets, and implements three main types of annotations, genomic mapping, mapping relation, and pathway enrichment with auxiliary functions. The package was open-designed and can be easily extended to broaden its application. We applied *snpGeneSets* to T2D data from the NHGRI GWAS catalog, a GWAS from dbGaP, and a GWES from GEO to demonstrate its utility in interpreting GWS results and enhancing the understanding of genetic effects.

## Supplementary Material

Supplemental Material

## References

[bib1] Auerbach R. K. Chen B. Butte A. J., 2013 Relating genes to function: identifying enriched transcription factors using the ENCODE ChIP-Seq significance tool. Bioinformatics 29(15): 1922–1924. 23732275 10.1093/bioinformatics/btt316 PMC3712221

[bib2] Barrett T. Troup D. B. Wilhite S. E. Ledoux P. Evangelista C., 2011 NCBI GEO: archive for functional genomics data sets–10 years on. Nucleic Acids Res. 39(Database issue): D1005–D1010. 21097893 10.1093/nar/gkq1184 PMC3013736

[bib3] Boyle A. P. Hong E. L. Hariharan M. Cheng Y. Schaub M. A., 2012 Annotation of functional variation in personal genomes using RegulomeDB. Genome Res. 22(9): 1790–1797. 22955989 10.1101/gr.137323.112 PMC3431494

[bib4] Croft D. Mundo A. F. Haw R. Milacic M. Weiser J., 2014 The reactome pathway knowledgebase. Nucleic Acids Res. 42(Database issue): D472–D477. 24243840 10.1093/nar/gkt1102 PMC3965010

[bib5] Eyre T. A. Ducluzeau F. Sneddon T. P. Povey S. Bruford E. A., 2006 The HUGO gene nomenclature database, 2006 updates. Nucleic Acids Res. 34(Database issue): D319–D321. 16381876 10.1093/nar/gkj147 PMC1347509

[bib6] Fabregat A. Sidiropoulos K. Garapati P. Gillespie M. Hausmann K., 2016 The reactome pathway knowledgebase. Nucleic Acids Res. 44(D1): D481–D487. 26656494 10.1093/nar/gkv1351 PMC4702931

[bib7] Fujita P. A. Rhead B. Zweig A. S. Hinrichs A. S. Karolchik D., 2011 The UCSC genome browser database: update 2011. Nucleic Acids Res. 39(Database issue): D876–D882. 20959295 10.1093/nar/gkq963 PMC3242726

[bib8] Geer R. C. Sayers E. W., 2003 Entrez: making use of its power. Brief. Bioinform. 4(2): 179–184. 12846398 10.1093/bib/4.2.179

[bib9] Harris M. A. Clark J. Ireland A. Lomax J. Ashburner M., 2004 The Gene Ontology (GO) database and informatics resource. Nucleic Acids Res. 32(Database issue): D258–D261. 14681407 10.1093/nar/gkh036 PMC308770

[bib10] Hirschhorn J. N., 2009 Genomewide association studies–illuminating biologic pathways. N. Engl. J. Med. 360(17): 1699–1701. 19369661 10.1056/NEJMp0808934

[bib11] Holmans P. Green E. K. Pahwa J. S. Ferreira M. A. R. Purcell S. M., 2009 Gene ontology analysis of GWA study data sets provides insights into the biology of bipolar disorder. Am. J. Hum. Genet. 85(1): 13–24. 19539887 10.1016/j.ajhg.2009.05.011 PMC2706963

[bib12] Kanehisa M. Goto S. Furumichi M. Tanabe M. Hirakawa M., 2010 KEGG for representation and analysis of molecular networks involving diseases and drugs. Nucleic Acids Res. 38(Database issue): D355–D360. 19880382 10.1093/nar/gkp896 PMC2808910

[bib13] Kanehisa M. Goto S. Sato Y. Furumichi M. Tanabe M., 2012 KEGG for integration and interpretation of large-scale molecular data sets. Nucleic Acids Res. 40(Database issue): D109–D114. 22080510 10.1093/nar/gkr988 PMC3245020

[bib14] Kinsella R. J. Kahari A. Haider S. Zamora J. Proctor G., 2011 Ensembl BioMarts: a hub for data retrieval across taxonomic space. Database (Oxford) 2011: bar030. 21785142 10.1093/database/bar030 PMC3170168

[bib15] Liberzon A. Subramanian A. Pinchback R. Thorvaldsdottir H. Tamayo P., 2011 Molecular signatures database (MSigDB) 3.0. Bioinformatics 27(12): 1739–1740. 21546393 10.1093/bioinformatics/btr260 PMC3106198

[bib16] Maglott D. Ostell J. Pruitt K. D. Tatusova T., 2011 Entrez gene: gene-centered information at NCBI. Nucleic Acids Res. 39(Database issue): D52–D57. 21115458 10.1093/nar/gkq1237 PMC3013746

[bib17] Marselli L. Thorne J. Dahiya S. Sgroi D. C. Sharma A., 2010 Gene expression profiles of beta-cell enriched tissue obtained by laser capture microdissection from subjects with type 2 diabetes. PLoS One 5(7): e11499. 20644627 10.1371/journal.pone.0011499 PMC2903480

[bib18] Mei H. Li L. Liu S. Jiang F. Griswold M., 2015 The uniform-score gene set analysis for identifying common pathways associated with different diabetes traits. BMC Genomics 16(1): 336. 25898945 10.1186/s12864-015-1515-3 PMC4415316

[bib19] Nam D. Kim J. Kim S.-Y. Kim S., 2010 GSA-SNP: a general approach for gene set analysis of polymorphisms. Nucleic Acids Res. 38 (Web Server issue): W749–W754. 20501604 10.1093/nar/gkq428 PMC2896081

[bib20] Peng G. Luo L. Siu H. Zhu Y. Hu P., 2010 Gene and pathway-based second-wave analysis of genome-wide association studies. Eur. J. Hum. Genet. 18(1): 111–117. 19584899 10.1038/ejhg.2009.115 PMC2987176

[bib21] Prentki M. Madiraju S. R., 2008 Glycerolipid metabolism and signaling in health and disease. Endocr. Rev. 29(6): 647–676. 18606873 10.1210/er.2008-0007

[bib22] Rung J. Brazma A., 2013 Reuse of public genome-wide gene expression data. Nat. Rev. Genet. 14(2): 89–99. 23269463 10.1038/nrg3394

[bib23] Scott L. J. Mohlke K. L. Bonnycastle L. L. Willer C. J. Li Y., 2007 A genome-wide association study of type 2 diabetes in Finns detects multiple susceptibility variants. Science 316(5829): 1341–1345. 17463248 10.1126/science.1142382 PMC3214617

[bib24] Segre A. V., DIAGRAM Consortium, MAGIC InvestigatorsGroop L. Mootha V. K., 2010 Common inherited variation in mitochondrial genes is not enriched for associations with type 2 diabetes or related glycemic traits. PLoS Genet. 6(8): e1001058. 10.1371/journal.pgen.1001058 PMC2920848 20714348

[bib25] Sherry S. T. Ward M. H. Kholodov M. Baker J. Phan L., 2001 dbSNP: the NCBI database of genetic variation. Nucleic Acids Res. 29(1): 308–311. 11125122 10.1093/nar/29.1.308 PMC29783

[bib26] Smyth G. K., 2004 Linear models and empirical Bayes methods for assessing differential expression in microarray experiments. Stat. Appl. Genet. Mol. Biol. 3(1): 3. 10.2202/1544-6115.1027 16646809

[bib27] Subramanian A. Tamayo P. Mootha V. K. Mukherjee S. Ebert B. L., 2005 Gene set enrichment analysis: a knowledge-based approach for interpreting genome-wide expression profiles. Proc. Natl. Acad. Sci. USA 102(43): 15545–15550. 16199517 10.1073/pnas.0506580102 PMC1239896

[bib28] Tryka K. A. Hao L. Sturcke A. Jin Y. Wang Z. Y., 2014 NCBI’s database of genotypes and phenotypes: dbGaP. Nucleic Acids Res. 42(1): D975–D979. 24297256 10.1093/nar/gkt1211 PMC3965052

[bib29] Visscher P. M. Brown M. A. McCarthy M. I. Yang J., 2012 Five years of GWAS discovery. Am. J. Hum. Genet. 90(1): 7–24. 22243964 10.1016/j.ajhg.2011.11.029 PMC3257326

[bib30] Wang K. Li M. Bucan M., 2007 Pathway-based approaches for analysis of genomewide association studies. Am. J. Hum. Genet. 81(6): 1278–1283. 17966091 10.1086/522374 PMC2276352

[bib31] Ward L. D. Kellis M., 2012 HaploReg: a resource for exploring chromatin states, conservation, and regulatory motif alterations within sets of genetically linked variants. Nucleic Acids Res. 40(Database issue): D930–D934. 22064851 10.1093/nar/gkr917 PMC3245002

[bib32] Welter D. MacArthur J. Morales J. Burdett T. Hall P., 2014 The NHGRI GWAS catalog, a curated resource of SNP-trait associations. Nucleic Acids Res. 42(Database issue): D1001–D1006. 24316577 10.1093/nar/gkt1229 PMC3965119

[bib33] Zambelli F. Prazzoli G. M. Pesole G. Pavesi G., 2012 Cscan: finding common regulators of a set of genes by using a collection of genome-wide ChIP-seq datasets. Nucleic Acids Res. 40(W1): W510–W515. 22669907 10.1093/nar/gks483 PMC3394253

