# Supplementary material for: snpGeneSets: An R Package for Genome-Wide Study Annotation
Source: G3 (Bethesda). 2016 Nov 2;6(12):4087–95. doi: 10.1534/g3.116.034694 (PMC5144977; doi:10.1534/g3.116.034694)
Supplement: Supplemental Material [file supp_6_12_4087__index.html]

snpGeneSets: An R Package for Genome-wide Study Annotation — snpGeneSets: An R Package for Genome-Wide Study Annotation — Supplemental Material 

# *snpGeneSets*: An *R* Package for Genome-Wide Study Annotation

## Supplemental Material for Mei *et al.*, 2016

**Files in this Data Supplement:**

- Figure S1 - Association of the number of genes with the number of gene-mapped SNPs. (.jpg, 902 KB)
- Figure S2 - ?log10(p-value) of KEGG gene sets from the USGSA (type 2) enrichment analysis. (.jpg, 1 MB)
- Table S1 - T2D SNPs identified in the GWAS catalog. (.xlsx, 41 KB)
- Table S2 - Relation mapping of GWAS risk SNPs to genes. (.xlsx, 18 KB)
- Table S3 - The type I analysis of KEGG gene sets enriched for T2D-mapped genes. (.xlsx, 22 KB)
- Table S4 - Genetic mapping annotation for component genes of the SPINK1 gene set. (.xlsx, 11 KB)
