# Supplementary figures and images for: snpGeneSets: An R Package for Genome-Wide Study Annotation
Source: G3 (Bethesda). 2016 Nov 2;6(12):4087–95. doi: 10.1534/g3.116.034694 (PMC5144977; doi:10.1534/g3.116.034694)

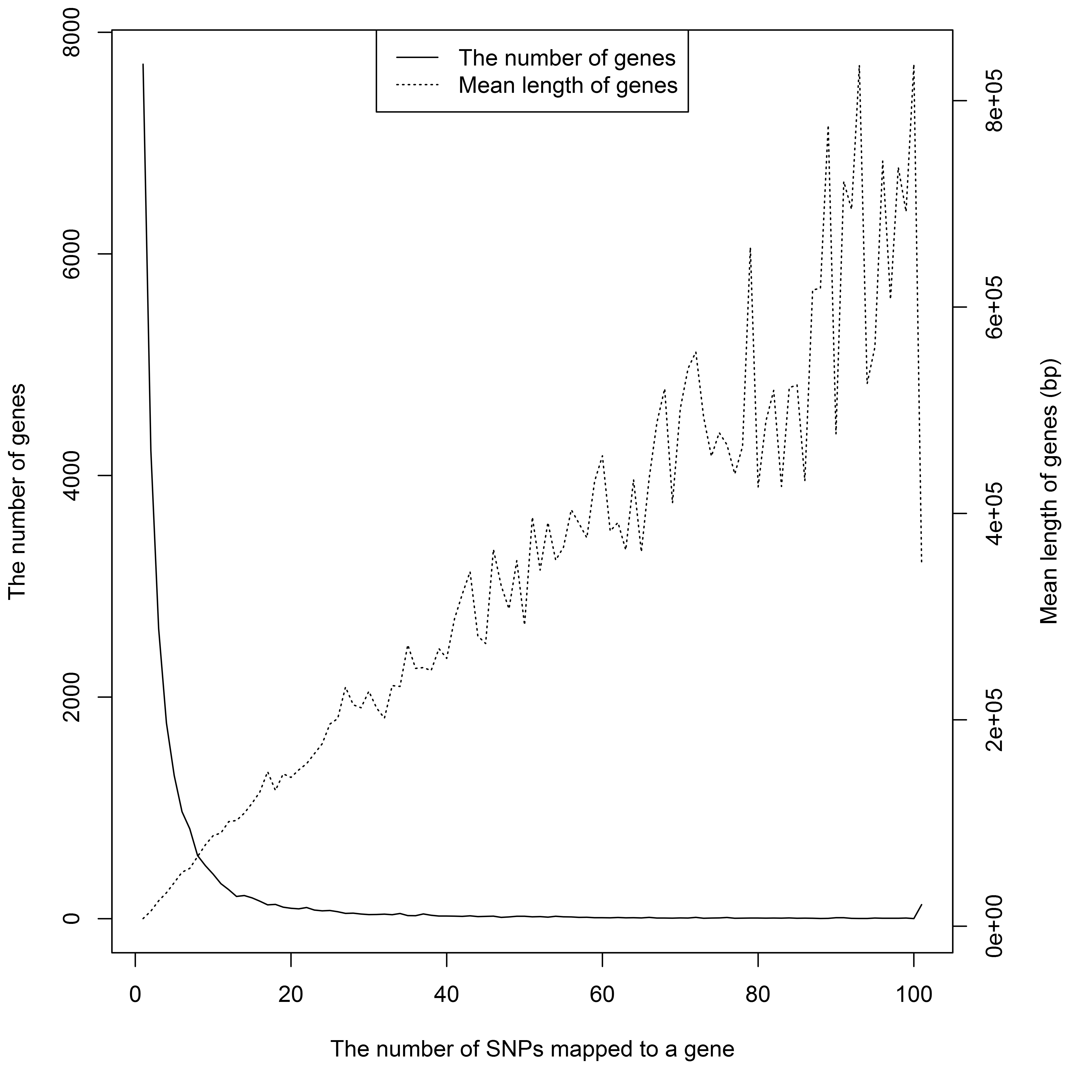

Supplement: Supplemental Material [file supp_g3.116.034694_FigureS1.jpg]

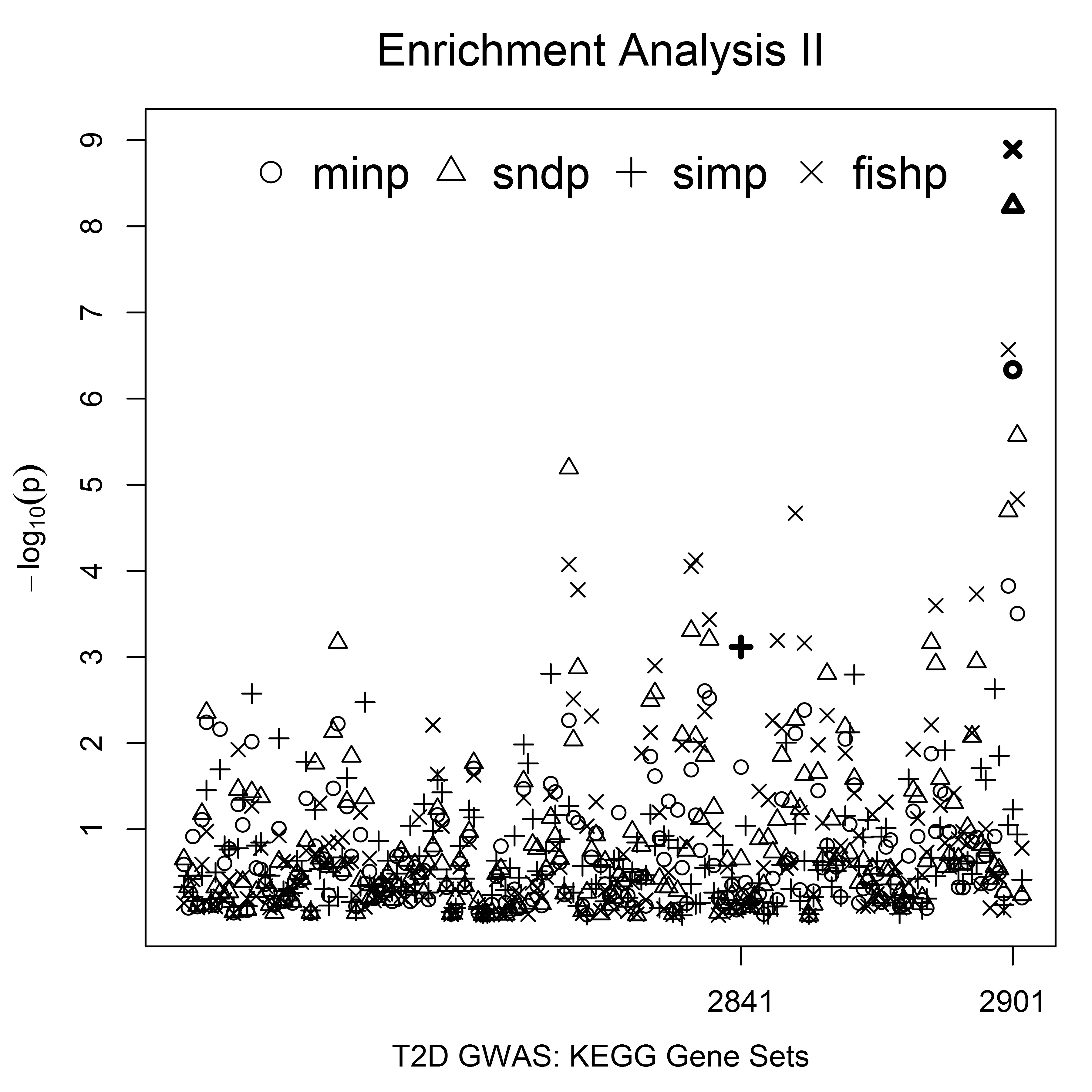

Supplement: Supplemental Material [file supp_g3.116.034694_FigureS2.jpg]
